# *Gelidium* Bioactives in Gut and Skin Homeostasis: Mechanisms, Microbiota Modulation, and the Gut–Skin Axis

**DOI:** 10.3390/microorganisms14092039

**Published:** 2026-09-12

**Authors:** Kyucheol Lee, Sang-Hoon Lee, Soohwan Jung, Kyu-Nam Kim

**Affiliations:** 1 Cheongdamrui Plastic Surgery, Jeju-si 63099, Republic of Korea; cdruilee@gmail.com; 2 Functional Medicine Clinic, Greencross I-MED Gangnam Center, Seoul 06223, Republic of Korea; shlee0612@naver.com; 3 Family Medicine, Philip Medical Center, Seongnam-si 13499, Republic of Korea; 2027fmlove@naver.com; 4 Department of Family Practice and Community Health, Ajou University School of Medicine, Suwon 16499, Republic of Korea

**Keywords:** agarose, agaro-oligosaccharides, bromophenols, mycosporine-like amino acids, prebiotics, short-chain fatty acids, photoaging, melanogenesis

## Abstract

The marine ecosystem remains an unparalleled reservoir of structurally unique macromolecular biomolecules with diverse pharmaceutical applications. Among red macroalgae (Rhodophyta), the genus *Gelidium* Lamouroux is economically renowned as a primary industrial source of agar. Beyond its traditional hydrocolloid utility, recent molecular evidence reveals that *Gelidium* biomass possesses a dense matrix of non-digestible sulfated galactans, low-molecular-weight agaro-oligosaccharides, marine bromophenols, polyphenols, and mycosporine-like amino acids that have shown promising biological activities and therapeutic potential in preclinical models. Utilizing advanced green depolymerization and extraction technologies—including enzyme-assisted, ultrasound-assisted, and subcritical water extraction—these structural polysaccharides and specialized secondary metabolites can be efficiently isolated while optimizing yields and preserving bioactive functional integrity. This review comprehensively synthesizes the dual-target therapeutic modalities of Gelidium bioactives, focusing on their prebiotic modulation of the gastrointestinal microbiota and their protective effects on cutaneous tissues. In the gastrointestinal tract, Gelidium polysaccharides resist upper digestive enzymatic hydrolysis, serving as selective fermentable substrates that significantly enrich beneficial saccharolytic taxa while suppressing opportunistic pathobionts. This microbial fermentation accelerates the synthesis of short-chain fatty acids; based on established epithelial models, these organic acids provide a mechanistic rationale for upregulating epithelial tight junction proteins (zonula occludens-1, occludin, and claudin-1), theoretically supporting intestinal barrier integrity and mitigating systemic endotoxemia. Concurrently, in cutaneous tissues, selected Gelidium-derived fractions have been reported to modulate matrix metalloproteinases, including matrix metalloproteinase-1, matrix metalloproteinase-2, and matrix metalloproteinase-9, scavenge reactive oxygen species via nuclear factor erythroid 2-related factor 2 and heme oxygenase-1 pathway activation, and modulate oxidative and melanogenic pathways to support cutaneous homeostasis. Finally, we establish the theoretical framework of the gut–skin axis as an experimentally supported systemic conduit through which Gelidium-mediated intestinal homeostasis attenuates cutaneous inflammation, photoaging, and barrier disruption. We further address current bioavailability limitations, standardization hurdles, and future clinical trajectories required to translate Gelidium biomass into functional nutricosmetics and marine therapeutics.

## 1. Introduction

The marine red algal genus *Gelidium* Lamouroux is economically renowned as a primary raw material for industrial agar extraction [1]. Beyond traditional hydrocolloid utility, recent molecular evidence indicates that *Gelidium* biomass contains a dense matrix of non-digestible sulfated galactans, low-molecular-weight agaro-oligosaccharides (AOS), bromophenols, polyphenols, and mycosporine-like amino acids (MAAs) [2]. Because human digestive enzymes cannot hydrolyze marine galactans, these polysaccharides pass into the large bowel to serve as selective prebiotic substrates [3]. Microbial fermentation of these glycans expands beneficial saccharolytic taxa and elevates short-chain fatty acids (SCFAs) to reinforce intestinal barrier integrity [4]. Concurrently, *Gelidium elegans* Kützing extracts have been documented to regulate mitogen-activated protein kinase (MAPK) and phosphoinositide 3-kinase/protein kinase B pathways, which modulate metabolic inflammation and attenuate downstream oxidative stress [5]. This review synthesizes these dual-target mechanisms, bridging prebiotic gut remodeling and dermal protection through the systemic framework of the gut–skin axis [6]. We evaluate current green extraction technologies, detail molecular signaling cascades, and outline translational perspectives for developing *Gelidium*-based nutricosmetics and marine therapeutics [7].

## 2. Literature Search Methodology

To ensure academic transparency, reproducibility, and rigorous data selection, this review combines elements of a narrative synthesis with a structured, systematic search framework. The literature search was executed across three primary electronic databases: PubMed/MEDLINE, Scopus, and the Web of Science Core Collection. The search timeline was limited to peer-reviewed articles published up to June 2026, with an emphasis on original research papers, molecular mechanistic studies, and high-impact reviews from the past ten years.

### 2.1. Search Strategy and Boolean Matrix

The electronic search utilized a precise combination of Medical Subject Headings (MeSH) terms and uncontrolled keywords. The primary search string was constructed using the following Boolean matrix: (“Gelidium” OR “Gelidium amansii” OR “Gelidium elegans” OR “Gelidium sesquipedale”) AND (“gut microbiota” OR “gut microbiome” OR “prebiotics” OR “short-chain fatty acids” OR “intestinal barrier” OR “skin” OR “photoaging” OR “matrix metalloproteinases” OR “melanogenesis” OR “tyrosinase” OR “atopic dermatitis” OR “gut-skin axis”).

### 2.2. Inclusion and Exclusion Criteria

To refine the body of literature, strict eligibility criteria were applied to the retrieved documents:

- Inclusion Criteria: (1) Peer-reviewed original research articles investigating the chemical isolation, extraction, or characterization of *Gelidium* species; (2) In vitro, in vivo (animal models), or clinical studies examining the physiological effects of *Gelidium* raw extracts, fractions, or isolated compounds on gastrointestinal parameters or cutaneous tissues; (3) Articles detailing the molecular signaling pathways (e.g., NF-κB, MAPK, MITF) activated or suppressed by *Gelidium* bioactives; (4) Academic literature explicitly discussing marine-derived compounds within the context of the gut–skin axis.
- Exclusion Criteria: (1) Non-peer-reviewed literature, including conference abstracts, patents, dissertations, and preprints; (2) Studies focusing exclusively on ecological, environmental, or purely agricultural cultivation aspects of *Gelidium* without biomedical, nutritional, or cosmeceutical applications; (3) Studies utilizing generic, non-identified “red seaweed extracts” where the specific inclusion of *Gelidium* could not be verified; (4) Articles published in languages other than English without available validated translations.

### 2.3. Screening Framework and PRISMA-Style Flow

The initial database interrogation yielded a total of 412 records. After executing automated and manual deduplication routines, 285 unique records remained. Phase I screening involved a blind, independent title and abstract review conducted by two independent reviewers, resulting in the exclusion of 142 records due to irrelevance or mismatch with the core inclusion criteria. Phase II screening entailed full-text evaluation of the remaining 143 articles. During this stage, 23 articles were excluded because they lacked specific molecular endpoints or did not isolate the *Gelidium* genus clearly. Ultimately, high-quality peer-reviewed articles were selected as the primary foundation for this comprehensive review, providing a robust, data-driven synthesis of the genus.

## 3. Chemical Composition of Gelidium

The clinical and cosmeceutical efficacy of *Gelidium* species is entirely dictated by the structural configuration and concentration of its diverse biomolecules. The cellular architecture of *Gelidium* can be divided into structural cell wall polysaccharides and specialized intracellular secondary metabolites. The global distribution, dominant bioactive fractions, and primary core molecular targets of major commercial *Gelidium* species are systematically collated in Table 1.

### 3.1. Structural Galactans: Agarose and Agaropectin

The principal chemical component of *Gelidium*, comprising up to 30–50% of its dry biomass, is agar, a high-molecular-weight hydrophilic galactan [10]. From a chemical perspective, agar is not a homogenous polymer but a complex mixture of two primary fractions: agarose and agaropectin [11].

Agarose represents the neutral, gelling fraction. It is a linear polymer characterized by alternating units of β-D-galactopyranose and 3,6-anhydro-α-L-galactopyranose linked via alternating β-(1 → 4) and α-(1 → 3) glycosidic bonds [12]. This specific stereochemical arrangement allows the polymer chains to spontaneously form stable, left-handed double helices at room temperature, aggregating into an insoluble three-dimensional microporous gel network.

In contrast, agaropectin is the non-gelling, acidic, and heavily modified fraction [11]. While it shares the same basic oligosaccharide backbone as agarose, it is randomly substituted with varying degrees of sulfate esters, D-glucuronic acid residues, and cyclic pyruvate ketals [13]. The presence of these negatively charged sulfate and carboxyl groups interrupts linear helical stacking, drastically reducing gelling capability and vastly enhancing its biochemical interactivity with human cellular membranes and the metabolic machinery of the gut microbiota.

### 3.2. Agaro-Oligosaccharides

While native agar possesses remarkable physical properties, its immense molecular weight severely restricts its absorption across cellular membranes and dampens its metabolic utility in biological systems [12]. Consequently, significant research has focused on AOS, which are low-molecular-weight degradation products generated through controlled chemical or enzymatic cleavage of *Gelidium* agarose [4]. AOS are categorized based on their terminal sugar residues. The agaro-series oligosaccharides possess a D-galactose residue at the reducing terminus, whereas the neoagaro-series oligosaccharides (NAOS) possess a 3,6-anhydro-L-galactose residue at the reducing terminus [14]. NAOS, particularly neoagarobiose, neoagarotetraose, and neoagarohexaose, exhibit superior water solubility, exceptional stability, and greater physicochemical accessibility, which may enhance their biological utilization by deep colonic microflora and facilitate their bioavailability.

### 3.3. Phenolic and Lipophilic Metabolites

Beyond structural carbohydrates, *Gelidium* synthesizes highly specialized secondary metabolites to neutralize the oxidative stress induced by continuous exposure to solar ultraviolet (UV) radiation and fluctuating saline concentrations in marine ecosystems [15]. Chief among these are marine bromophenols and polyphenolic compounds [2]. Bromophenols isolated from *Gelidium* feature benzene rings substituted with bromine and hydroxyl groups, a unique configuration that imparts exceptional electron-donating properties [2]. Additionally, *Gelidium* contains functional lipophilic pigments, including carotenoids (predominantly lutein and beta-carotene) as well as chlorophyll-a, all of which possess intrinsic radical-scavenging capabilities [15].

### 3.4. Phycobiliproteins and Mycosporine-like Amino Acids

The intracellular matrix of *Gelidium* is highly enriched with phycobiliproteins, which serve as accessory light-harvesting water-soluble proteins [16]. These include Phycoerythrin, which gives the algae its vibrant red hues, and Phycocyanin [16]. Structurally, these proteins contain covalently bound open-chain tetrapyrrole chromophores (bilins) that act as potent, non-toxic free radical terminators [16]. Furthermore, like many Rhodophyta species, *Gelidium* contains low-molecular-weight MAAs, including shinorine and porphyra-334 derivatives [17]. Structurally composed of a cyclohexenone or cyclohexenimine chromophore conjugated with amino acid residues, MAAs absorb ultraviolet radiation across the UVA and UVB ranges (310–360 nm) and dissipate photochemical energy thermally, serving as biological photoprotectants against solar irradiation [17].

## 4. Extraction Technologies

To isolate these intracellular and structural biomolecules from the tough, fibrillar cell wall matrix of *Gelidium* without inducing structural denaturation, conventional organic solvent extraction has been rapidly superseded by green, energy efficient, and sustainable extraction platforms [7]. A comprehensive technological comparison of conventional versus advanced green extraction parameters and efficiencies for *Gelidium* biomass is provided in Table 2.

### 4.1. Enzyme-Assisted Extraction

Enzyme-Assisted Extraction (EAE) employs carbohydrate-active enzymes under mild conditions (typically 40–50 °C and physiological pH) to selectively disrupt macroalgal biomass [19]. In this approach, cellulases and related cell-wall-degrading enzymes facilitate matrix breakdown to release intracellular phycobiliproteins and phenolics, whereas specialized marine agarases (exo- and endo-agarases) specifically depolymerize the agarose backbone into bioactive agaro-oligosaccharides. The principal advantage of EAE lies in its high catalytic specificity, which avoids harsh organic solvents, prevents toxic byproduct formation, and preserves delicate functional groups such as ester sulfates.

### 4.2. Ultrasound-Assisted Extraction

Ultrasound-Assisted Extraction (UAE) leverages the physical phenomenon of acoustic cavitation [18]. When high-frequency ultrasound waves ranging from 20 to 100 kHz pass through an aqueous extraction solvent containing *Gelidium* biomass, they induce the rapid formation, growth and violent collapse of microscopic cavitation bubbles [21]. This implosion generates localized micro-jets, severe shear forces, and shockwaves that mechanically fracture thick macroalgal cell walls. UAE drastically accelerates mass transfer, reduces extraction time from hours to minutes, minimizes solvent consumption, and allows for the highly efficient extraction of thermolabile polyphenols, bromophenols, and pigments without thermal degradation.

### 4.3. Subcritical Water Extraction

Subcritical Water Extraction (SWE), also designated as pressurized hot water extraction, operates at elevated temperatures (typically between 100 °C and 250 °C) and sufficient pressures to maintain water in its liquid state [20]. Under subcritical conditions, the dielectric constant (ε) of water drops significantly, mimicking the properties of moderately polar organic solvents [22]. This allows water to efficiently dissolve both polar polysaccharides and non-polar lipophilic phenolics simultaneously. Furthermore, the auto-ionization of subcritical water yields an increased concentration of hydronium ions ($H_{3}O^{+}$), initiating a controlled auto-hydrolysis that cleaves large agar chains directly into bioavailable agaro-oligosaccharides within a single operational sequence.

## 5. Gut Microbiota Modulation

The upper gastrointestinal tract of humans lacks specialized endogenous glycoside hydrolases required to cleave the equatorial β-glycosidic bonds of agarose or modified links of agaropectin [12]. Consequently, *Gelidium*-derived polysaccharides and oligosaccharides function as true dietary prebiotics, passing unaltered through the stomach and small intestine to undergo extensive colonic fermentation by large bowel microflora [4].

**Figure 1:**
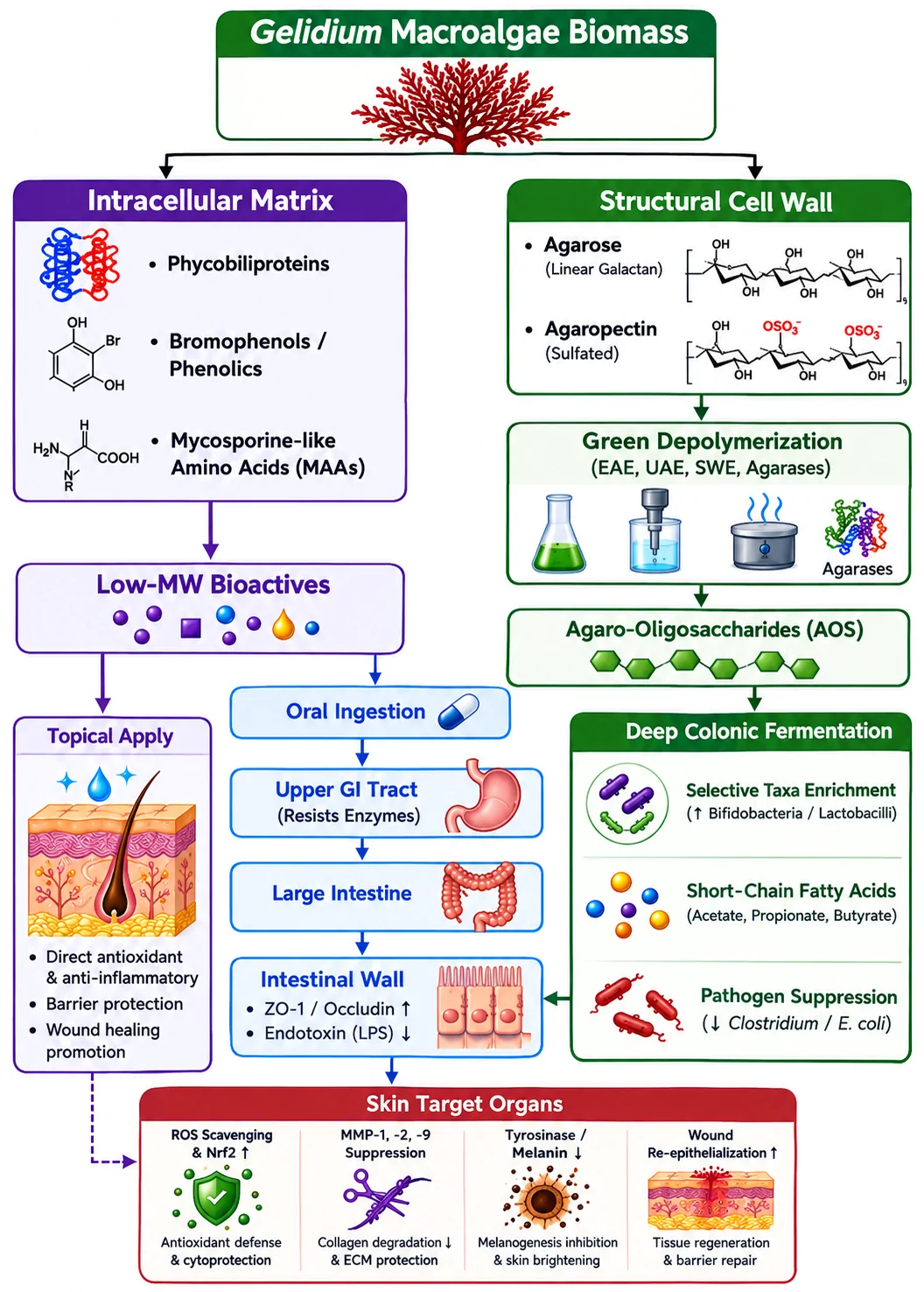
Proposed mechanisms underlying the beneficial effects of *Gelidium*-derived bioactive compounds on skin health through the gut–skin axis. Schematic overview illustrating the major bioactive constituents of *Gelidium* and their proposed pathways contributing to skin health. Intracellular bioactive compounds, including phycobiliproteins, bromophenols, phenolic compounds, and mycosporine-like amino acids, together with structural polysaccharides (agarose and agaropectin), are extracted from *Gelidium* biomass. Green depolymerization technologies generate low-molecular-weight agaro-oligosaccharides, which are resistant to upper gastrointestinal digestion and undergo selective fermentation by the colonic microbiota. This process promotes beneficial bacterial populations, enhances short-chain fatty acid production, suppresses pathogenic microorganisms, and strengthens intestinal barrier integrity by upregulating tight junction proteins while reducing lipopolysaccharide translocation. Bioactive metabolites subsequently enter systemic circulation and contribute to skin homeostasis through antioxidant, anti-inflammatory, anti-photoaging, anti-melanogenic, and wound-healing activities. Direct topical application of *Gelidium*-derived bioactives may additionally exert local protective effects on the skin. The proposed mechanisms are synthesized from current experimental evidence and represent potential biological pathways rather than fully established causal relationships. In the diagram, solid arrows represent directly demonstrated experimental pathways, whereas dashed arrows denote proposed or indirect mechanistic connections within the gut–skin framework. EAE, enzyme-assisted extraction; ECM, Extracellular matrix; Low-MW, Low-molecular-weight; LPS, Lipopolysaccharide; GI, gastrointestinal; MMP, matrix metalloproteinase; SWE, subcritical water extraction; UAE, ultrasound-assisted extraction; ZO-1, zonula occludens-1.

To illustrate the systemic implications of this process, the proposed mechanisms underlying the beneficial effects of *Gelidium*-derived bioactive compounds on skin health through the gut–skin axis are summarized in Figure 1.

### 5.1. Taxonomic Remodeling of the Microbial Consortium

Molecular profiling using 16S rRNA next-generation sequencing has confirmed that the administration of *Gelidium* raw polysaccharides and AOS induce a major taxonomical remodeling of the intestinal architecture [4]. *Gelidium* bioactives function as selective carbon and energy sources for specific beneficial saccharolytic bacteria that express specialized agarases or possess robust cross-feeding networks. The specific relative abundance shifts and host physiological impacts driven by *Gelidium* prebiotics are detailed in Table 3.

Regarding the enrichment of *Bifidobacterium* spp., these beneficial saccharolytic taxa undergo significant numerical expansion within the colonic microflora upon *Gelidium* prebiotic supplementation [23]. Mechanistically, these microorganisms utilize saccharolytic pathways to ferment macroalgal oligosaccharide fragments, contributing to the increased production of beneficial organic acids [23]. Furthermore, clinical supplementation with *Gelidium*-derived AOS has been shown to selectively expand and activate agarose- and AOS-utilizing gut taxa, inducing specialized carbohydrate-active enzymes that facilitate glycan degradation and cross-feeding networks [4]. Concurrently, *Gelidium* fermentation contributes to the suppression of potential pathobionts, particularly limiting the overgrowth of facultative anaerobic bacteria such as *Escherichia coli* [23]. Furthermore, *Gelidium* intake is associated with a modulation of the Firmicutes/Bacteroidetes (F/B) ratio, potentially supporting metabolic homeostasis through altered dietary carbohydrate utilization [23].

### 5.2. SCFAs Biosynthetic Kinetics

The principal mechanism through which the *Gelidium*-modulated microbiota influences host physiology is the substantial increase in SCFAs, predominantly acetate, propionate, and butyrate [24]. When beneficial colonic taxa ferment *Gelidium* AOS, they break down the galactan backbone into pyruvate via the glycolytic pathway, which is subsequently converted into volatile organic acids [25]. The intestinal synthesis yields, host cellular receptors, and tissue-specific therapeutic outcomes of these main SCFAs are outlined in Table 4.

Among these organic acids, butyrate acts as the definitive primary oxidative fuel for host colonocytes, supporting ATP production and cellular health [29]. Meanwhile, propionate migrates via the portal vein to the liver, where it acts as an inhibitor of hepatocyte gluconeogenesis and cholesterol synthesis [27]. Concurrently, acetate enters systemic arterial circulation, modulating peripheral tissue lipid profiles and serving as an important signaling ligand for metabolic regulation [26].

### 5.3. Intestinal Barrier Reinforcement and Anti-Endotoxemia Mechanisms

The multi-fold increase in local SCFA concentrations directly drives structural preservation of the intestinal mucosal barrier [28]. Butyrate binds to G-protein coupled receptors (GPR41 and GPR43) expressed on the basolateral membrane of the intestinal epithelium, activating downstream intracellular pathways that significantly upregulate the transcription and assembly of tight junction proteins, specifically Zonula Occludens-1 (ZO-1), occludin, and claudin-1 [30].

General studies on marine and dietary polysaccharides suggest that reinforcing paracellular tight junctions against macromolecular toxins like lipopolysaccharide (LPS) provides a plausible mechanistic rationale for attenuated endotoxemia [31]. Within this context, *Gelidium* prebiotic fermentation is proposed to support systemic inflammatory homeostasis via SCFA-mediated barrier protection. Supporting local intestinal benefits, *Gelidium* fractions have been demonstrated to suppress intestinal nuclear factor-kappa B (NF-κB) activation, downregulating mucosal expression of tumor necrosis factor-alpha (TNF-α), interleukin-1 beta (IL-1β), and interleukin-6 (IL-6) [32].

## 6. Skin Health

Cutaneous architecture undergoes continuous degenerative degradation driven by intrinsic chronological factors and extrinsic photoaging, which is heavily mediated by solar ultraviolet (UVA/UVB) radiation [33]. *Gelidium* bioactives exert targeted protective effects within both dermal and epidermal layers [8]. The downstream cellular targets, pathways, and visible dermatological manifestations of isolated *Gelidium* bioactives are summarized in Table 5.

### 6.1. Cellular Antioxidant Cascades via Nrf2/HO-1

UV exposure strikes epidermal keratinocytes and dermal fibroblasts, inducing rapid intracellular accumulation of Reactive Oxygen Species (ROS), such as singlet oxygen (O21), superoxide anions ($\cdot O_{2}^{-}$), and hydrogen peroxide ($H_{2}O_{2}$) [36]. These radicals initiate lipid peroxidation of cellular membranes and break down nuclear DNA [37]. Polyphenols, bromophenols, and protein/phycobiliprotein-rich fractions isolated from *Gelidium sesquipedale* (Clemente) have been demonstrated to counteract oxidative stress via direct radical scavenging, wherein phenolic hydroxyl groups and bilin chromophores neutralize free radicals and inhibit oxidative chain reactions [9]. In broader cellular physiology, endogenous cytoprotection against sustained oxidative stress relies heavily on the Nrf2/ARE signaling pathway [38]. Under oxidative stimulation, Nrf2 dissociates from Keap1 and translocates into the nucleus to induce cytoprotective enzymes, including heme oxygenase-1 (HO-1), superoxide dismutase (SOD), catalase (CAT), and glutathione peroxidase (GPx) [38]. While direct *Gelidium*-mediated Nrf2/ARE transactivation has been characterized in metabolic models [5], its specific role in dermal cytoprotection is proposed as a biologically plausible mechanism supported by the radical-scavenging capacity of red algal secondary metabolites [9,39].

### 6.2. Inhibition of Matrix Metalloproteinases and Extracellular Matrix Preservation

Dermal aging is characterized by structural collapse of the extracellular matrix (ECM), composed primarily of Type I and Type III collagens and elastic fibers [40]. Elevated ROS levels activate MAPKs—including ERK, JNK, and p38 MAPK [41]. This signaling cascade triggers the activation of transcription factor Activator Protein-1 (AP-1), which upregulates the transcription of Matrix Metalloproteinases (MMPs) [42]. MMP-1 initiates cleavage of structural fibrillar collagens, whereas MMP-2 and MMP-9 degrade fragmented collagen fragments and basement membrane elastin [43]. Bioactive fractions and sulfated polysaccharides isolated from *Gelidium* species directly block these degenerative pathways. Specifically, sulfated polysaccharides from *Gelidium crinale* (Hare ex Turner) Gaillon significantly downregulate the expression of MMP-9 [34], while bioactive extracts from *Gelidium amansii* attenuate UVB-induced MAPK phosphorylation, effectively suppressing MMP-1 expression and preventing collagen fragmentation [8]. This regulatory mechanism preserves dermal matrix density and attenuates wrinkle formation in preclinical photoaging models.

### 6.3. Anti-Melanogenic Pathways and Enzymatic Tyrosinase Blockade

Hyperpigmentation disorders stem from hyperactivation of melanogenesis within epidermal melanocytes [44]. Tyrosinase is the key rate-limiting enzyme that catalyzes the hydroxylation of L-tyrosine to L-DOPA, and subsequent oxidation of L-DOPA to DOPAquinone [45]. Phenolic- and bromophenol-enriched extracts from *Gelidium corneum* (Hudson) J.Agardh exhibit notable photoprotective, antioxidant, and potential anti-melanogenic properties in skin cellular models [2]. Although in vitro assays demonstrate modulation of oxidative stress and melanin-related pathways, detailed molecular investigations into direct enzymatic tyrosinase inhibition and regulatory cascades remain an area for further validation [2].

### 6.4. Wound Healing Acceleration and Anti-Dermatitis Activity

*Gelidium*-derived polysaccharides form stable, cross-linked biomimetic hydrogels that maintain optimal moisture retention across wounded cutaneous tissue [46]. These macroalgal matrices have been demonstrated to modulate the microenvironment of human dermal fibroblasts, thereby facilitating skin repair, controlling angiogenesis, and accelerating granulation tissue assembly [47]. In inflammatory dermatoses, systemic or topical application of *Gelidium* extracts effectively targets epidermal cellular pathways, significantly suppressing hyper-activated inflammatory responses and protecting human keratinocytes from tissue damage [35]. Consistently, in vivo models have confirmed that treatment with *Gelidium amansii* extracts suppress systemic inflammatory responses, downregulate pro-inflammatory cytokines, and attenuate tissue-specific metabolic inflammatory deviations [33].

## 7. Gut–Skin Axis

The bidirectional physiological link between the gut and the skin, known as the gut–skin axis, serves as a primary conduit through which oral consumption of marine algal functional components—such as dietary fibers and polysaccharides found in Gelidium—translate into visible cutaneous benefits [48]. This complex communication network is maintained via three interconnected pathways, categorized as immunological, metabolic, and neuroendocrine signaling axes.

### 7.1. Immunological Linkage and Systemic Inflammatory Reset

Under dysbiotic conditions, heightened mucosal permeability facilitates systemic translocation of gut-derived LPS and pro-inflammatory pathobionts into circulation [48]. This systemic endotoxemia triggers a host immune cascade: circulating LPS binds to Toll-Like Receptor 4 on peripheral immune cells and cutaneous dendritic cells, driving downstream activation of the pro-inflammatory NF-κB signaling network [49]. Consequently, elevated systemic levels of pro-inflammatory cytokines (including IL-1β, IL-6, and TNF-α) reach dermal compartments, prompting fibroblasts and keratinocytes to overexpress MMPs, accelerating ECM breakdown and exacerbating inflammatory skin disorders.

While orally administered *Gelidium*-derived polysaccharides and agaro-oligosaccharides have been demonstrated to selectively enrich beneficial taxa (*Bifidobacterium*) and elevate SCFA yields in experimental models [23,30], their capacity to blunt downstream systemic LPS translocation and cutaneous inflammation is proposed as a comprehensive mechanistic framework informed by general gut–skin axis biology [48,49].

While much of the mechanistic framework governing the gut–skin axis relies on preclinical animal paradigms, a growing body of human clinical trials supports the translational validity of targeted dietary microbial modulation. In broader clinical settings, targeted prebiotic and synbiotic interventions promoting colonic *Bifidobacterium* have been shown to reduce disease severity and serum inflammatory biomarkers in patients with atopic dermatitis [50]. Additionally, non-digestible marine-derived glycans can attenuate low-grade mucosal inflammation and circulating endotoxins in human subjects [51]. Although dedicated human dermatological trials specifically utilizing *Gelidium* biomass remain to be conducted, these findings provide supportive clinical evidence for the broader concept that intestinal microbiome modulation may influence systemic inflammatory homeostasis, but *Gelidium*-specific clinical evidence for skin outcomes remains to be established.

### 7.2. Metabolic Translocation of Active Metabolites

Beyond immunological control, metabolic byproducts of *Gelidium* colonic fermentation are distributed systemically to drive dermal homeostasis [48]. Ingested *Gelidium* polysaccharides escape upper gastrointestinal digestion to undergo colonic fermentation, which has been demonstrated to significantly increase total SCFA production [23]. General physiological literature provides supporting evidence that circulating SCFAs reach the skin via cutaneous microvasculature to modulate local immune homeostasis [48]. Although direct *Gelidium*-specific cutaneous tracing remains an area of ongoing investigation, this metabolic translocation is proposed as a key systemic conduit through which oral algal bioactives support dermal resilience. Furthermore, low-molecular-weight phenolic metabolites and MAAs co-absorbed in the gut are distributed throughout dermal tissues, where they provide ongoing systemic protection against UV-induced oxidative stress and matrix degradation [2].

## 8. Therapeutic Applications

The unique chemical properties of *Gelidium* enable diverse therapeutic applications spanning nutritional, medical, and cosmeceutical industries.

### 8.1. Functional Nutricosmetics and Dietary Supplements

The integration of *Gelidium* biomass into functional foods and oral nutraceuticals directly aligns with the evolving paradigm of “beauty-from-within.” Oral formulations containing enzymatically degraded *Gelidium* agaro-oligosaccharides serve as systemic anti-aging agents that leverage gut microbial fermentation to improve cutaneous biophysical parameters. Comprehensive academic syntheses indicate that the systematic ingestion of marine-derived prebiotic oligosaccharides effectively orchestrates the gut–skin axis to optimize cutaneous physiology. Mechanistically, these non-digestible glycan fractions promote a systemic homeostatic reset that correlates with a marked reduction in transepidermal water loss, secondary to enhanced stratum corneum hydration and the structural preservation of dermal microrelief networks [52].

Parallel clinical evidence highlights systemic multi-organ benefits of oral *Gelidium* ingestion, particularly in modulating gut-metabolic homeostasis. In 12-week RCTs involving overweight and obese individuals, daily administration of *Gelidium elegans* extract (1000 mg/day) significantly reduced total body weight and visceral fat area [53], while simultaneously alleviating functional bowel symptoms and abdominal discomfort [54]. Additionally, short-term dietary supplementation with agaro-oligosaccharides (200 mg/day) selectively enriched colonic agarose-utilizing taxa with high gastrointestinal tolerability [4]. Together, these translational human findings confirm that orally administered *Gelidium* bioactives may have potential as dual-target functional candidates, capable of improving gastrointestinal parameters while offering preclinically supported cutaneous benefits through the gut–skin axis.

### 8.2. Topical Cosmeceuticals and Formulation Matrix

In the cosmeceutical field, *Gelidium*-derived extracts have been increasingly investigated for use in topical formulations, including anti-photoaging preparations, photoprotective agents, and barrier-supporting matrices [2]. Their UV-absorbing MAAs function as stable, non-toxic bio-sunscreens, replacing synthetic chemical UV filters that can cause skin irritation or environmental damage [2]. Additionally, the film-forming properties of *Gelidium* agarose serve as an exceptional natural vehicle, improving skin penetration and sustained release of other hydrophilic actives [55].

### 8.3. Biomedical Devices and Advanced Wound Dressings

In regenerative medicine, *Gelidium* agar-derived hydrogels are engineered into advanced biocompatible wound dressings [56]. These hydrogels can be infused with bioactive bromophenols or antimicrobial agents to create a sterile, responsive wound environment [57]. Their high porosity and water-holding capacity facilitate efficient absorption of wound exudates while supporting continuous gas exchange, promoting rapid tissue repair in chronic wounds [58]. To systematically evaluate the translation of these multi-systemic mechanisms from bench to bedside, the current state of scientific evidence regarding *Gelidium* bioactives and gut–skin axis components is consolidated in Table 6.

## 9. Challenges and Future Perspectives

Despite the clear therapeutic potential of *Gelidium* species, several critical biochemical, technical, and regulatory hurdles must be systematically resolved before full commercialization can be realized.

### 9.1. Bioavailability and Molecular Weight Optimization

The foremost challenge lies in the high molecular weight and complex structural cross-linking of native *Gelidium* polysaccharides, which limit their solubility and direct bioavailability [59]. Future research must prioritize optimization of green, sequence-specific extraction methodologies—particularly cloning and industrial scale-up of recombinant endo-agarases—to reliably produce agaro-oligosaccharides with precise degrees of polymerization (DP 4 to 6), maximizing both prebiotic fermentation and skin penetration.

### 9.2. Standardization and Quality Control Metrology

As wild marine organisms, *Gelidium* populations exhibit substantial seasonal, geographical, and environmental variations in chemical composition [60]. A wild cohort harvested in the Atlantic may present an altered sulfate or polyphenol profile compared to a Pacific cohort [61]. Establishing standardized quality control parameters, utilizing high-performance liquid chromatography-mass spectrometry fingerprinting, and setting strict baseline metrics for active marker compounds are required to ensure therapeutic consistency [62].

### 9.3. Heavy Metal Bioremediation and Aquaculture Safety

Because marine macroalgae naturally bioaccumulate environmental toxins, wild-harvested *Gelidium* carries an inherent risk of contamination with toxic heavy metals, including inorganic arsenic, cadmium, lead, and mercury [63,64]. To guarantee consumer safety and meet strict international pharmaceutical guidelines, future industrial production should transition away from wild harvesting toward highly controlled Integrated Multi-Trophic Aquaculture frameworks [65]. These land-based or coastal aquaculture systems allow for precise regulation of water purity, nutrient input, and light exposure, ensuring sustainable cultivation of clean, ultra-pure *Gelidium* biomass. Established maximum allowable thresholds, analytical metrologies, and core rationales for regulatory compliance are defined in Table 7.

### 9.4. Rigorous Human Clinical Validation

Although initial clinical trials in humans have provided promising evidence regarding gut and metabolic benefits, well-designed, large-cohort, randomized, double-blind, placebo-controlled trials specifically evaluating skin-targeted parameters and metagenomic profile shifts remain limited. Future research trajectories must focus on clinical validation, utilizing advanced metagenomic sequencing and cutaneous bioengineering tools to confirm the therapeutic efficacy of *Gelidium* bioactives in human populations.

## 10. Conclusions

*Gelidium* Lamouroux species represent a promising marine biomaterial at the intersection of biotechnology, gastroenterology, and dermatology. This review synthesized the genus along five core domains: chemical profiling, green extraction, gut microbiota modulation, cutaneous protection, and the integrated gut–skin axis. Structurally, *Gelidium* yields non-digestible sulfated galactans, bioavailable AOS, antioxidant bromophenols, and photoprotective MAAs. Advanced green extraction platforms, including enzyme-assisted, ultrasound-assisted, and subcritical water extraction, offer complementary strategies for recovering polysaccharides, oligosaccharides, and selected secondary metabolites, although operational severity must be carefully optimized to minimize the thermal degradation of heat-sensitive compounds.

Mechanistically, *Gelidium* AOS and galactans resist upper digestion to selectively nourish colonic saccharolytic taxa (e.g., *Bifidobacterium*), significantly increasing SCFA production.

While *Gelidium* bioactives have been demonstrated to nourish beneficial saccharolytic taxa and downregulate local inflammatory cytokines, the subsequent upregulation of tight junction proteins (ZO-1, occludin, claudin-1) and attenuation of systemic LPS translocation represent a proposed, biologically plausible axis grounded in the SCFA literature. Systemically, these findings provide a plausible mechanistic rationale by which improved intestinal barrier integrity and attenuated systemic inflammatory signaling could support dermal homeostasis; however, direct *Gelidium*-specific gut–skin causal evidence remains limited and requires dedicated clinical validation.

Rather than a fully established clinical solution, *Gelidium* offers an experimentally supported strategy for systemic health via the gut–skin axis. To translate these findings into medical and nutricosmetic practice, future trajectories must focus on standardizing active marker compounds, scaling up sequence-specific recombinant endo-agarase enzymatic processing, and conducting rigorous, large-cohort human RCTs.

## Figures and Tables

**Table 1:** Global distribution and primary biomedical activities of key *Gelidium* species.

Algal Species	Primary Geographical Habitat	Dominant Bioactive Fractions	Core Biomedical Focus	Target Molecular Endpoints	References
*Gelidium amansii* J.V.Lamouroux	Coastal East Asia (Korea, Japan, China)	Fermented Extracts, Bioactive Polysaccharides	Dermal Photoprotection, Anti-photoaging, Collagen Integrity	Suppression of UVB-induced MAPK activation and MMP-1 expression	[8]
*Gelidium elegans* Kützing	Western Pacific Intertidal Zones	Polyphenols, Bromophenols, Agarose Matrix	Metabolic Regulation, Antioxidant Defense, Gut-Adipose Axis	Regulation of MAPK and PI3K/Akt signaling, activation of Nrf2/HO-1	[5]
*Gelidium sesquipedale* (Clemente) Thuret	Eastern Atlantic (Spain, Portugal, Morocco)	Polyphenolic Fractions, Proteins, Bioactive Lipids	Endogenous Antioxidant Support, Cytoprotection	Direct ROS scavenging, prevention of oxidative macromolecular damage	[9]
*Gelidium corneum* (Hudson) J.Agardh	Mediterranean Sea, North Atlantic	Polyphenols, Bromophenols, Mycosporine-like Amino Acids (Shinorine)	Biological UV Protection, Photoprotection, Dermal Support	Modulation of oxidative and melanogenic pathways; UVA/UVB photoprotection (310–360 nm)	[2]

Low-MW, Low Molecular Weight; MMP-1, Matrix Metalloproteinase-1; NF-κB, Nuclear Factor Kappa B; Nrf2/HO-1, Nuclear Factor Erythroid 2-Related Factor 2/Heme Oxygenase-1; ROS, Reactive Oxygen Species; UVA/UVB, Ultraviolet A/Ultraviolet B.

**Table 2:** Comparison of conventional vs. advanced green extraction methodologies for *Gelidium*.

Extraction Technology	Operational Characteristics	Extraction Efficiency	Bioactive Structural Integrity	Industrial Scale-up Considerations	Environmental Profile	References
Conventional Hot Water	Moderate temperature; relatively long extraction time	Moderate; established agar recovery	Potential degradation of heat-sensitive compounds during prolonged heating	High energy demand and prolonged processing	Higher energy consumption	[18]
Enzyme-Assisted (EAE)	Mild conditions with selective enzymatic hydrolysis	Enhanced recovery of selected target fractions	Generally favorable under mild conditions	Enzyme cost and process optimization remain challenges	Reduced severity of processing conditions	[19]
Ultrasound-Assisted (UAE)	Acoustic cavitation; shortened extraction time	Enhanced extraction efficiency	May improve recovery while reducing thermal exposure	Scale-up may be limited by acoustic energy distribution	Reduced extraction time and solvent demand	[18]
Subcritical Water (SWE)	Pressurized hot water; reduced dependence on organic solvents	Potentially high recovery of selected compounds	Dependent on temperature and pressure; excessive severity may promote degradation	Requires pressure-resistant equipment	Reduced use of organic solvents	[20]

Low-MW, Low Molecular Weight; pH, Potential of Hydrogen; kHz, Kilohertz.

**Table 3:** Microbiota changes associated with *Gelidium*-derived prebiotics.

Target Microbial Taxa	Reported Change	Potential Microbial Mechanism	Potential Host-Relevant Effect	References
*Bifidobacterium* spp.	Increased	Utilization of agar-derived oligosaccharides and saccharolytic fermentation	Increased production of microbial metabolites, including SCFAs	[23]
Agarose/AOS-utilizing gut bacteria	Increased/activated	Carbohydrate-active enzymes involved in agarose/AOS degradation	Enhanced microbial carbohydrate utilization and cross-feeding	[4]
*Escherichia coli*	Decreased	Alteration of the luminal environment associated with increased saccharolytic fermentation	Potential reduction in expansion of facultative anaerobic bacteria	[23]
Firmicutes/Bacteroidetes ratio	Altered/normalized	Changes in microbial carbohydrate utilization	Potential association with metabolic effects	[23]

AOS, Agaro-Oligosaccharides; SCFAs, Short-Chain Fatty Acids.

**Table 4:** Proposed SCFAs-mediated molecular mechanisms potentially linked to *Gelidium* prebiotic fermentation.

SCFAs	Major Microbial SCFAs Product	Primary Host Molecular Targets	Intestinal Mechanism of Action	Potential Skin-Relevant Effects	References
Acetate	Major microbial fermentation product	GPR43, GPR41	Metabolic signaling and peripheral energy regulation	Potential contribution to epithelial and metabolic homeostasis	[26]
Propionate	Major microbial fermentation product	GPR43, GPR41	Modulation of hepatic gluconeogenesis through GPR43/AMPK signaling	Potential immunomodulatory effects; direct skin-specific evidence remains limited	[27]
Butyrate	Major microbial fermentation product	GPR41, GPR43, HDACs	Supports colonocyte metabolism and intestinal epithelial barrier function through tight-junction regulation	Potential anti-inflammatory effects; direct *Gelidium*-specific skin evidence remains limited	[28]

AMPK, AMP-Activated Protein Kinase; GPR, G-Protein Coupled Receptor; HDAC, Histone Deacetylase; SCFAs, Short-Chain Fatty Acids.

**Table 5:** Molecular targets and cutaneous effects of Gelidium-derived bioactive fractions.

Bioactive Fraction/Compound(s)	Experimental Skin/Cell Model	Primary Molecular Target/Pathway	Direct Molecular or Enzymatic Effect	Reported Cutaneous/Cellular Outcome	References
Sulfated Polysaccharide Fraction (*Gelidium crinale*)	Human fibrosarcoma cells (HT1080)	NF-κB, MAPK, mTOR/PI3K/Akt	Reduced MMP-9 mRNA, expression and activity	Reduced cell migration/invasion and MMP-9-associated matrix degradation	[34]
Bioactive Polyphenolic/Polysaccharide Extract (*Gelidium amansii*)	Human dermal fibroblasts; hairless mouse skin	UVB-associated photoaging pathways	Reduced MMP-1 expression; increased type I procollagen	Reduced wrinkle formation and epidermal thickening; improved skin hydration	[8]
Phenolic- and Bromophenol-Enriched Fraction (*Gelidium corneum*)	HaCaT keratinocytes; RAW 264.7 macrophages; mouse skin formulation model	Oxidative and inflammatory responses	Antioxidant/photoprotective activity	Photoprotection, antioxidant activity and wound-healing potential	[2]
Phycobiliprotein-Rich Protein Fraction (*Gelidium sesquipedale*)	Cell-free antioxidant/enzymatic assays	Oxidative-stress-related targets	Radical-scavenging and anti-enzymatic activities	Potential dermocosmetic applications	[9]
Cellulose Nanocrystals (*Gelidium amansii*)	HaCaT keratinocytes; mouse skin	AP-1/MAPK and COX-2 signaling	Suppression of c-Jun/AP-1 activity and COX-2 expression	Reduced UVB-induced epidermal thickening and skin inflammation	[35]

AP-1, Activator Protein 1; COX-2, Cyclooxygenase-2; HaCaT, Human adult low-calcium high-temperature keratinocytes; HT1080, Human fibrosarcoma cell line; MAPK, Mitogen-Activated Protein Kinase; MMP, Matrix Metalloproteinase; mTOR, Mechanistic Target of Rapamycin; NF-κB, Nuclear Factor Kappa B; PI3K/Akt, Phosphoinositide 3-Kinase/Protein Kinase B; RAW 264.7, Murine macrophage cell line; UVB, Ultraviolet B.

**Table 6:** Levels of evidence and methodological characterization of investigations on *Gelidium* bioactives and the gut–skin axis.

Core Physiological Target	Bioactive Class/Candidate	Experimental Model(s)	Evidence Category *	Key Molecular/Cellular Mechanisms	References
Prebiotic Gut Remodeling	Low-MW Agaro-oligosaccharides (AOS/NAOS) (from *Gelidium pacificum*/*Gelidium* spp.)	In vitro human fecal fermentation; in vivo murine models	Preclinical (In vivo/In vitro)	Exerts a selective prebiotic effect by stimulating host saccharolytic fermentation pathways, shifting the gut microbiota toward a high-SCFAs-producing profile and blunting pathobiont expansion	[23]
Intestinal Barrier Reinforcement	Colonic-derived SCFAs (mechanistic proxy for *Gelidium* prebiotic fermentation)	In vitro Caco-2 monolayers; in vivo rodent models of colitis	Mechanistic Proxy (In vivo/In vitro)	General marine glycan models demonstrate that SCFAs upregulate tight junction proteins (ZO-1, occludin, claudin-1) via GPCR activation, providing a mechanistic rationale for suppressed systemic LPS translocation.	[31]
Dermal Photoprotection & ECM Integrity	Low-MW AOS/Extracts (from *Gelidium amansii*)	UVB-irradiated murine models; primary human dermal fibroblasts	Preclinical (In vivo/In vitro)	Attenuates MAPK (ERK/JNK/p38) phosphorylation and AP-1 activation; downregulates transcription of matrix metalloproteinases (MMP-1, MMP-2, MMP-9).	[8]
Endogenous Cytoprotection	Phycobiliprotein and Polyphenolic Fractions (from *Gelidium sesquipedale*)	In vitro cell-free radical assays; cellular antioxidant models	Mechanistic (In vitro/Cell-free)	Direct radical scavenging, termination of oxidative chain reactions, and macromolecular protection	[9]
Anti-Melanogenesis	Phenolic- and Bromophenol-Enriched Extracts (from *Gelidium corneum*)	In vitro B16F10 melanoma cells; cell-free enzymatic tyrosinase assays	Mechanistic (In vitro/Cell-free)	Demonstrates antioxidant and photoprotective modulation with potential anti-melanogenic activity; direct enzymatic tyrosinase inhibition remains a candidate mechanism under investigation	[2]
Anti-Dermatitis Activity	Cellulose Nanocrystals (from *Gelidium amansii*)	In vitro HaCaT human keratinocytes In vivo skin-inflamed mouse models	Preclinical (In vivo/In vitro)	Suppresses hyper-activated inflammatory responses via the downregulation of MAPK and COX-2 pathways; attenuates epidermal thickening and cutaneous tissue damage.	[35]
Wound Healing Acceleration	Sulfated Polysaccharides (from *Gelidium crinale*)	In vitro dermal fibroblasts In vivo skin excision/repair mouse models	Preclinical (In vivo/In vitro)	Promotes cutaneous tissue repair by modulating dermal fibroblast functions; regulates angiogenesis and fibrosis during granulation tissue assembly.	[47]
Gut Microbiota & Inflammatory/Metabolic Modulation	*Gelidium elegans* Extract (1000 mg/day), Targeted Prebiotic Formulations	Double-blind, randomized, placebo-controlled human trials	Clinical (Human RCT)	Significantly decreases total body weight, fat mass, and visceral fat; alleviates functional bowel symptoms (abdominal discomfort); reduces systemic mucosal inflammation.	[53,54]

AOS, Agaro-oligosaccharides; AP-1, Activator Protein 1; Caco-2, Human colorectal adenocarcinoma cell line; COX-2, Cyclooxygenase-2; ECM, Extracellular Matrix; ERK, Extracellular Signal-Regulated Kinase; GPCR, G-Protein Coupled Receptor; HaCaT, Human adult low-calcium high-temperature keratinocytes; JNK, c-Jun N-terminal Kinase; Low-MW, Low Molecular Weight; LPS, Lipopolysaccharide; MAPK, Mitogen-Activated Protein Kinase; MMP, Matrix Metalloproteinase; NAOS, Neoagaro-Oligosaccharides; RCT, Randomized Controlled Trial; SCFAs, Short-Chain Fatty Acids; UVB, Ultraviolet B; ZO-1, Zonula Occludens-1. * Note: Evidence categories classify the methodological design of the supporting literature, spanning human randomized controlled trials (Clinical), preclinical animal and cellular models (Preclinical/Mechanistic), and established biological proxies for gut–skin translocation pathways.

**Table 7:** Representative Quality Assurance and Safety Specifications for Gelidium Extracts.

Quality Assurance Parameter	Representative Regulatory Limits * & Guidelines	Primary Analytical Metrology	Core Rationale for Safety Regulation	References
Inorganic Arsenic	<1.0–3.0 ppm (ICH Q3D/Ph. Eur. herbal monographs)	ICP-MS	Highly carcinogenic; macroalgae are natural bioaccumulators	[63,64]
Lead & Cadmium	Pb < 0.5–3.0 ppm/Cd < 0.2–1.0 ppm (ICH Q3D/EU Reg. standards)	Atomic Absorption Spectroscopy/ICP-MS	Nephrotoxic and neurotoxic profiles; strict pharmaceutical caps	[63,64]
Total Pesticide Residues	Below LOQ/Zero Detection (USP <561>/Ph. Eur. 2.8.13)	GC-MS/MS, LC-MS/MS	Eliminates agricultural run-off contamination from coastal harvesters	[62]
Microbial Bioburden	TAMC < 10^3^ CFU/g; TYMC < 10^2^ CFU/g (Ph. Eur. 5.1.4/USP <61>)	Standard Plate Colony Enumeration	Ensures microbiological quality and safety for topical and oral applications	[62]

CFU/g, Colony-Forming Units per Gram; GC-MS/MS, Gas Chromatography–Tandem Mass Spectrometry; ICH, International Council for Harmonisation (Guideline Q3D: Elemental Impurities); ICP-MS, Inductively Coupled Plasma Mass Spectrometry; LC-MS/MS, Liquid Chromatography–Tandem Mass Spectrometry; LOQ, Limit of Quantification; Ph. Eur., European Pharmacopoeia; ppm, Parts Per Million; TAMC, Total Aerobic Microbial Count; TYMC, Total Yeast and Mold Count; USP, United States Pharmacopeia. * Note: Regulatory limits presented are representative/illustrative values based on pharmacopoeial monographs and ICH Q3D guidelines; applicable thresholds depend on the finished product category, jurisdiction, daily intake, and route of administration.

## Data Availability

No new data were created or analyzed in this study. Data sharing is not applicable to this article.
